# Von Hippel-Lindau is Associated to Pancreatic Neuroendocrine Tumors: A Comprehensive Review

**DOI:** 10.15586/jkcvhl.v10i2.272

**Published:** 2023-05-23

**Authors:** Danilo Coco, Silvana Leanza

**Affiliations:** 1 Department of General Surgery, AST 1 Pesaro-Urbino, Pesaro, Italy; 2 Department of General Surgery, Carlo Urbani Hospital, AST 2, Jesi (Ancona), Italy

**Keywords:** pancreatic neuroendocrine tumors (pNETs), surgery, Von Hippel-Lindau (VHL)

## Abstract

Multiorgan tumors are a hallmark of the autosomal dominant genetic disorder known as Von Hippel-Lindau syndrome (VHL), which is typically the result of inherited aberrations of the VHL tumor suppressor gene. The most frequent cancer is retinoblastoma, which can also occur in the brain and spinal cord, renal clear cell carcinoma (RCCC), paraganglioma, and neuroendocrine tumors. There may also be lymphangiomas, epididymal cysts, and pancreatic cysts or pancreatic neuroendocrine tumors (pNETs). The most frequent causes of death are metastasis from RCCC and neurological complications from retinoblastoma or central nervous system (CNS). Pancreatic cysts are present in 35–70% of VHL patients. Simple cysts, serous cysts, or pNETs are possible presentations, and the likelihood of malignant degeneration or metastasis is no greater than 8%. Although VHL has been associated with pNETs, their pathological characteristics are unknown. Furthermore, it is unknown whether variations in the VHL gene cause the development of pNETs. Hence, this retrospective study was undertaken with the main aim to examine whether pNETs are connected to VHL from a surgical perspective.

## Introduction

Von Hippel-Lindau (VHL) disease is a hereditary syndrome linked to the development of multiorgan neoplasms (1, 2). The incidence is roughly 1 in 36,000 live births (3). This disease is caused by the VHL tumor suppressor gene, which can be found on the short arm of chromosome 3 (1). This gene, which encodes the tumor suppressor protein pVHL, has three exons. Because of VHL gene mutations (mostly deletions), the absence of the pVHL promotes angiogenesis and unrestrained cell proliferation. The pVHL mutations consequently increase the risk of tumor development in the designated organs (4). Symptoms usually appear in the second decade of life. Women typically live to be 50 years old, compared to men who typically live to be 60. Complications from CNS and RCCC tumors lead to VHL-related death (5–7). In addition to cancers of the CNS, it is distinguished by multiorgan cancers of the kidney, pancreas, adrenal gland, and reproductive organs. Retinal or cerebral hemangioblastoma is the most common neoplasm, although spinal hemangioblastomas, RCCC, pheochromocytomas (Pheo), paragangliomas, pNETs, cystadenomas of the epididymis, and tumors of the lymphatic sac can also be found. About 70% of the cases of VHL (RCC) are RCCC and hemangioblastomas. There is a correlation between VHL and pNETs in these patients, with incidence rates ranging from 5 to 18% (3, 8, 9). The pathological characteristics of pNETs in VHL have not been described. Furthermore, it is unknown if variations in the VHL gene cause the development of pNETs. de Mestier et al. and Krauss et al., claim that VHL-related pNETs typically do not function and hardly ever cause disease symptoms (10, 11). However, these tumors can behave malignantly, and pNETs continue to be a major contributor to the morbidity and mortality linked to VHL (10–13). Furthermore, Ehrlich et al. found that VHL-associated pNETs frequently have multiple foci and generally advance more subtly. As a result, the VHL population is not covered by recommendations for diagnosis and treatment based on sporadic pNETs (14). Hence, this retrospective study was carried out with the main goal to examine whether pNETs are connected to VHL from a surgical perspective.

## Materials and Methods

Retrospective analysis of PubMed articles was done. When looking for VHL, 6537 results were returned. The number of results was drastically decreased by choosing only the articles from 2000 to 2021. The search was conducted using a combination of medical subject heading (MeSH) terms and keywords. VHL and pNETs were the inclusion requirements. The database subject terms were taken from MeSH and Emtree. Reviews and case studies were not included. Only articles written in English, Dutch, French, and German were available. There was no restriction based on the publication year. “VHL surgical treatment” served as the main outcome indicator. The articles were examined by two authors. The articles used randomized controlled trials (RCTs), and cohort, case-control, and randomized studies, as well as prospective and retrospective studies as their methodologies. All linguistic studies were included. Case reports and articles not devoted to surgical management were disregarded. Studies had to include a minimum of five patients suspected with pNETs, who had either a clinical or genetic diagnosis of VHL. Patients with no family history of VHL disease with the presence of two or more CNS hemangioblastomas or one CNS hemangioblastoma and one visceral tumor (renal and epididymal cysts) were excluded from the study. The patient satisfies the diagnostic requirements for VHL if clear cell renal carcinoma, pheochromocytoma, or hemangioblastoma was discovered and data extraction was feasible. After entering all identified articles into Rayyan QCRI, duplicate articles were removed. In accordance with the aforementioned inclusion criteria, two reviewers (S.A. and M.R.N.) independently read full texts of any potentially relevant articles after screening study titles and abstracts for all studies. For full-text articles, the reasons for exclusion were noted.

### Identification

A systematic search was conducted in PubMed to identify relevant studies on VHL-associated pNETs. The search was performed using a combination of MeSH terms and keywords, with VHL and pNETs as inclusion requirements.

### Screening

The titles and abstracts of the identified studies were screened to exclude irrelevant studies. Reviews and case studies were also excluded.

### Eligibility

Full-text articles were evaluated for eligibility. Studies that met the inclusion criteria and had a minimum of five patients with suspected pNETs who had either a clinical or genetic diagnosis of VHL were included.

### Included

After removing duplicates and applying inclusion criteria, the studies that met the criteria were included in the analysis.

### Excluded

Studies that did not meet the inclusion criteria, were duplicates, or were not in English, Dutch, French, or German language were excluded.

### Quality assessment

The quality of the included studies was evaluated using the relevant quality assessment tools.

### Data extraction

Data were extracted from the included studies using a standardized data extraction form.

### Synthesis

The data from the included studies were synthesized and analyzed to draw conclusions.

## Results

Lubensky et al. examined 14 pNETs histopathologically in VHL patients (15). Additionally, fluorescence in situ hybridization and single-strand conformational polymorphism analysis based on polymerase chain reaction were used to examine DNA from pNETs and healthy pancreatic tissue in 6 of the 14 patients with documented germ-line VHL gene mutations. The tumors have distinct bands of stromal collagen and a solid, molecular and/or glandular structure that give them their distinctive morphology. The tumors in 60% of cases had at least focal clear cell cytology. The endocrine immunohistochemical markers chromogranin A and/or synaptophysin were positive in all tumors; pancreatic polypeptides, somatostatin, insulin, and/or glucagon were localized positively in 35% of pNETs; and pancreatic and gastrointestinal hormone immunity were absent in 65% of tumors. When examined under an electron microscope, dense nerve granules were visible, and the cells also amply demonstrated an abundance of lipids in the cytoplasm. Genetic examination of all pNETs revealed an allele loss of the second copy of the VHL gene. They established the pNET as a distinct tumor type of the VHL disease by demonstrating the presence of VHL allelic deletion genes, which provides evidence for the role of genes in tumorigenesis. According to Libutti et al., there was a significant difference (P = 0.0013) in the size of the primary tumor between patients with hepatic metastases (median 5 cm; range 3–8 cm) and patients without them (median 2 cm). Patients without metastatic disease had tumors smaller than 3 cm. They suggested yearly CT/MRI surveillance for tumors less than 1 cm and individual risk assessments for tumors between 1 and 3 cm for metastases in light of these findings. Resection is recommended for tumors that are >2 cm in size, symptomatic, producing hormones, growing, or >3 cm in size when discovered in the head of the pancreas. Libuti et al. implemented the aforementioned guidelines. The 25 surgical patients did not have any metastatic disease (15). A second study by Marcos et al., which found that none of the 18 tumors under 3 cm had metastatic disease, corroborated these findings from the observation that only two of the 11 tumors larger than 3 cm were malignant (16). According to Davenport et al., none of the five patients who underwent surgical resection for six pNETs ranging in size from 0.3 to 2.1 cm had any signs of metastatic disease. The diagnosis showed a pNET of 8.5 cm in the single patient with metastatic disease (17). de Mestier et al. contrasted the long-term effects of sporadic nonmetastatic pNETs and those brought on by VHL in their study (10). We compared VHL patients who underwent resection for a pNET to surgical patients with a matched number of sporadic pNETs. The tumors of the remaining 11 patients were not resected if they were smaller than 1.5 cm, but 12 patients had all of their pNETs removed. After a 10-year follow-up, none of these tumors showed signs of progressive disease. These results are in favor of managing small pNETs in an organ-sparing way. Tumor recurrence occurred in two VHL patients with pNETs larger than 50 mm. The Blansfield et al. study included 108 VHL patients with pNETs. There was confirmed metastatic disease in nine patients. In eight patients, the primary tumor was located in the head of the pancreas. Primary pNETs larger than 3.0 cm in size were present in six patients. Two patients died as a result of complications brought on by metastases (18). On the other hand, the primary tumor size was greater than 3.0 cm in 19 patients who did not have metastatic disease. Fifty five patients with metastatic disease were shown in Krauss et al. report (11). The maximum tumor diameter of metastatic pNETs (median 5 cm; range 2.8–7.0) and nonmetastatic pNETs (median 2 cm; range 0.4–10) differed significantly (P 0.001) from each other. The smallest pNET in this cohort with metastatic disease measured 2.8 cm. No matter where the tumor is located, the maximum tumor diameter is the best indicator of metastatic disease (PPV 51%, NPV 100% for 2.8 cm diameter cut-off). 117 patients with pNETs larger than 1.5 cm underwent surgery. All patients who underwent surgery had a significantly longer 10-year survival rates but those with pNETs smaller than 2.8 cm benefited the most. Thus, Krauss et al. suggested that annual monitoring for tumors that approach a diameter of 2 cm and surgery should be strongly considered for pNETs above 2.5 cm (11). The study by Corcos et al (13). also used the previous resection standards of Libutti et al. (2000) reportings (15). The largest pNETs in 11 patients with lymph node metastases ranged from 1.5 to 8.0 cm, while those in 6 patients with liver metastases ranged from 1.5 to 8.0 cm. Five of the patients with metastatic pNETs had tumors smaller than 2.6 cm, and one metastatic pNET measured 1.5 cm. Last but not least, no progressive disease was found during the follow-up period (median 5 years; range 1–10) for the five patients who had small tumors that were not removed. These findings suggest that the criteria may be too lax and that smaller tumors should be operated (Tables 1 and 2).

**Table 1:** Summarizing the papers selected with reference, number of patients, and outcomes.

References	Number of patients	Outcome
(10)	Not specified	Patients with VHL diesease who undergo surgery to remove pancreatic neuroendocrine tumors (PNETs) hava a favorable long-term prognosis. Smaller PNETs left in place did not progress, and a surgery strategy that spares the parenchyma seems appropriate for VHL-PNETs.
(11)	Not specified	There is no specific medical therapy for VHL-PNETs. Surveillance is recommended for small, asymptomatic discovered PNETs and surgical resection is indicated for symptomatic or growing tumors.
(13)	23 patients with VHL disease and PNETs	Patients with VHL disease and PNETs may have multiple tumors that can be nonfunctional and located in the pancreatic head, body, and tail.
(15)	12 patients with VHL disease and PNETs	Most PNETs associated with VHL disease are nonfunctional, small and slow-growing tumors that are more frequently located in the pancreatic tail.
(11)	Not specified	Imaging studies are helpful in diagnosing PNET.

**Table 2:** Common presentation, criteria for surgery, routine manegement, and outcome.

Common presentation	Criteria for surgery	Routine management	Outcome
Pancreatic Neuroendocrine Tumors (pNETs)	Size > 2 cm or functional tumors	Surveillance with imaging every 6-12 months	Surgical resection is the treatment of choice, with a 5-year survival rate of up to 94%
Hemangioblastomas	Symptomatic or > l cm	Observation with imaging every 6-12 months	Surgical resection is indicated in symptomatic or large hemangioblastomas. The 5-year survival rate for patients undergoing resection is up to 97%
Renal Cell Carcinoma (RCCC)	Size > 3 cm or rapidly growing tumors	Surveillance with imaging every 6-12 months	Surgical resection is the primary treatment for RCCC. with a 5-year survival rate of up to 95%
Pheochromocytomas (Pheo)	Symptomatic or > 5 cm	Medical management with alpha and beta blockade, followed by surgical resection	The 5-year survival rate for patients undergoing surgical resection is up to 95%

Patients with VHL require long-term monitoring and management due to the risk of developing multiple neoplasms. Regular imaging is necessary to detect and evaluate the size and growth of tumors. Surgical intervention is recommended for tumors that meet specific criteria, including size, symptoms. and functionality. The overall prognosis for patients with VHL is good with early detection and appropriate management.

## Discussion

VHL is currently categorized clinically into the 3 kinds. Type 2 exhibits three of these subtypes. Renal cell carcinoma poses a significant risk for type 1. The traits of Types 2a and 2b have high risk of renal cell carcinoma and pheochromocytoma, whereas the trait of Type 2c has the high risk of pheochromocytoma. Simple cysts, serous cystadenomas, mixed neuroendocrine tumors, pNETs, and simple cysts are the pancreatic lesions that are seen in VHL disease. The majority of cases (17–72%) involve cysts. pNETs 2 cm in diameter or larger, or pNETs of any size with malignant characteristics, must undergo pancreatectomy. If there are no histopathological signs of malignant tumors, radiological follow-up can be used to detect small tumors. Excision is performed if the pancreatic NET is small (8). The European-American-Asian-VHL-panNET-Registry was the largest cohort reporting pNET penetrance (11). This registry included 2330 VHL patients in total, and 273 (12%) of them had pNETs. A 10-year-old patient who had nonmetastatic disease was the youngest. The average age of pNET onset (interquartile range: 10–75 years) was 35. No associations between genotype and age of onset were discovered. Before age 11, the authors suggest starting surveillance. Data from additional international cohorts show that pNET frequencies varied from 14 to 33%. The median age at which pNET first appeared was between 33 and 38 years, with a range of 12–68 years (18–20). pNETs found in the body and tail of the pancreas are treated with a distal pancreatectomy. Only the tail of the pancreas can be surgically removed, or a pancreatic splenectomy can be carried out using open, laparoscopic, or robotic surgery. pNETs found in the head of the pancreas are treated using the Whipple procedure. Small, low-grade tumors can be treated with a central pancreatectomy (8, 21, 22). Pancreatic cysts are present in between 35 and 70% of VHL patients. With a less than 8% chance of malignant degeneration or metastasis, these conditions can present as simple cysts, serous cysts, or pNETs. Resection criteria included a 3-cm tumor, a pathogenic variant in exon 3, and a tumor that had doubled in size in less than 500 days. Depending on the site, nucleation, Whipple, or distal pancreatectomy is advised because these characteristics raise the risk of metastasis (8, 18, 23, 24). The only reports on pNETs in VHL patients are case reports and radiological series. Despite the fact that there is evidence connecting pancreatic NETs to VHL disease, neither a pathological analysis of several tumors nor a look at genetic alterations in VHL-associated NETs have been published. 13% of the 256 VHL patients examined by the imaging tests conducted at the National Cancer Institute had solid pancreatic lesions, according to the results.The VHL patients with pNETs in this study had a mean age of 35 years, younger than the sporadic patients with pNETs (58 years). There was no hormonal activity in any tumor. Multiple pNETs that ranged in size from microscopic to macroscopic and were dispersed throughout the pancreas were frequently present in patients with VHL (15, 25–30). Despite typically being well contained within the pancreas, VHL-associated pNETs have the potential to spread elsewhere. Patients with metastases had a median primary tumor diameter of 5 cm versus a median primary tumor diameter of 2 cm in patients without metastatic disease. As a result, it seems that the size of the primary VHL, pNET, and the risk of metastatic disease are related (15, 30). All pancreatic lesions connected to VHL had pNET-like architecture on a histological level. Neuroendocrine differentiation was verified using the markers synaptophysin, chromogranin A, NSE, and S100, as well as the tiny, dense-core granules that could be seen under an electron microscope. One distinguishing features of VHL and pNETs was the presence of clear-cell morphology, which was present in 60% of all tumors, regardless of their sizes. The cytoplasmic clearing was attributed to prominent lipid globules and myelin that could only be seen under an electron microscope. Only a small portion of pNETs connected to VHL was found to have cytoplasmic glycogen on PAS and PAS-D stains or on electron microscopy. Metastatic renal cell carcinoma and pancreatic microcystic adenoma are the most frequent lesions to consider in the differential diagnosis of VHL and pNETs. In patients with VHL, solid microcystic adenomas can be challenging to distinguish from clear-cell pNETs, and both microcystic adenomas and NETs can arise in the same pancreas (15, 31). Two additional, peculiar pancreatic tumors could also be considered. The first clear-cell pancreatic “sugar” tumor that exhibits abundant cytoplasmic glycogen is HMB-45 positive, and is also cytokeratin, NSE, and chromogranin A negative, similar to “sugar” tumors of the lung (27). The second condition, pancreatic clear-cell carcinoma, has significant pleomorphism and invasiveness and is extremely rare (27, 32). The discovery of both pheochromocytomas and pNETs in VHL patients suggests that the disorder may be a continuum of multiple endocrine neoplasia. Multiple pNETs are common in multiple endocrine neoplasia type 1 (MEN1). Although there are some similarities between MEN1 and VHL disease, there are also some key differences in the pancreatic pathology of these patients. First of all, pNETs are found in 82–100% of MEN1 patients, while the incidence of VHL disease is 12–17%. Second, MEN1 typically has a higher proportion of hormonally functional NETs than the uncommon VHL disease (15, 27, 30, 33, 34). The discovery of allelic deletions of the VHL gene in pancreatic NETs establishes NET as a distinct tumor type of VHL disease and provides direct molecular evidence for the involvement of the VH gene, according to Knudson et al., which predicts that each neoplasm should have a genotype consisting of one allele with an inherited germ-line mutation and loss of the second wild-type allele, which happen through chromosomal deletion. However, given the lack of additional VHL patients with pancreatic carcinoma and the persistence of heterozygosity in adenocarcinoma, it is unlikely that VHL gene alterations contribute to the development of pancreatic adenocarcinoma in VHL patients. Despite being a highly sensitive imaging modality, endoscopic ultrasound (EUS) is less effective as a surveillance modality due to its operator dependence and invasive procedure (35). As a result, we suggest using either CT or MRI as the primary imaging technique, and only using EUS in cases where CT and/or MRI are inconclusive. 68Ga-DOTATATE PET/CT is comparable to and may even outperform CT and MRI in terms of detecting lesions. The use of this modality should be considered when examining patients who are thought to have metastatic disease. Because it is also used in treatment plans involving peptide receptor–targeted radiotherapy or somatostatin analogs, we advise 68Ga-DOTATATE PET/CT over 18F-FDG PET/CT because it is also incorporated into treatment plans that use somatostatin analogs or radiotherapy that targets peptide receptors. F-FDG PET/CT is a good backup because not all institutions may have access to 68Ga-DOTATATE PET/CT, especially for spotting metastases. The use of SRS, 18C-5-HTP PET, 11F-FDOPA, or pNET screening in VHL is not supported by any evidence (36, 37). The newest recommendations state that screening should start at age 12 (38). The VHL Alliance recommendations, however, advise that screening should start at age 16. Although there is no enough data to provide specific advice, monitoring should start before the age of 16. It is preferred to use individualized surveillance based on VHL mutation (39). The natural course of the tumor will be taken into account for the best surveillance. Due to a lack of data on the tumor growth of VHL-related pNETs, no recommendations for the ideal timing of pNETs can be made. Two studies reported metastatic disease in multiple patients with a pNET of 3.0 cm, despite the fact that several of the included studies found no metastatic disease in patients with a pNET of 3.0 cm. We therefore draw the conclusion that benign behavior is not always assured by a small tumor size (10, 11, 13, 15). According to Blansfield et al., patients with metastatic disease were more likely to have VHL exon 3 mutations (18). Based on the most recent findings from the largest VHL cohort found in the literature reported by Krauss et al. (11), we advise a cautious approach with yearly monitoring for tumors that approach a diameter of 2 cm. This strategy is in line with the current management strategy for sporadic nonfunctioning pNETs, which found that the majority of pNETs should be treated with a wait-and-see approach for tumors smaller than 2 cm in diameter (37). Given that earlier intervention was found to result in better long-term outcomes, surgical intervention for VHL-related pNETs smaller than 2.5 cm should be considered seriously (11, 37). Weisbrod et al. prospectively assessed the tumor growth of 163 pNETs in 87 VHL patients in just one study. The majority of growths in patients showed a slow growth rate and a nonlinear pattern. 20% of these tumors were stable on subsequent imaging, and 20% of them shrank on their own (40, 41). In a cohort of 108 VHL patients with pNETs reported by Bransfield et al., the tumor doubling time was discovered to be shorter in patients with metastases (median 337 days; range 180–463 days) than in patients without metastases (median 2630 days; range 103–9614 days, P 0.0001) (18).

Belzutifan, an HIF-2α inhibitor, has shown promising results in treating advanced forms of pheochromocytoma or paraganglioma, PNETs, and VHL disease–associated tumors in clinical trials (1). A case report also suggests exceptional activity in metastatic PNET arising from VHL with belzutifan (2). Meanwhile, somatostatin analogs, such as octreotide and lanreotide, have been shown to have antiproliferative effects in nonfunctional PNETs and may have a role in the management of VHL-associated PNETs (3). Further research is needed to determine the optimal use of these targeted therapies in the management of VHL-associated tumors (42–44).

## Conclusions

VHL syndrome has a wide range of symptoms and is frequently challenging to diagnose. It is a multisystem tumor syndrome that frequently affects the CNS as well as different internal organs. Patients with VHL frequently require multidisciplinary care from specialists in surgery, radiology, pathology, and oncology as well as genetic counselling. Early intervention and decreased morbidity and mortality result from early mutation identification. VHL is a rare disease, so there has not been much research on the management of this condition. Based on the most recent literature, we sought to provide evidence-based recommendations that would supplement the existing management strategies for use in clinical practice.
